# Comprehensive Tuberculosis Screening and Treatment at a Prison in Central Papua Province, Indonesia

**DOI:** 10.3390/tropicalmed9100241

**Published:** 2024-10-12

**Authors:** Aurelia, Kamaludin, Cahya Muslimin, Yetty Balik, Trisasi Lestari, Firdaus Hafidz, Christa Dewi, Christopher Lowbridge, Ari Probandari

**Affiliations:** 1 Papuan Health and Community Development Foundation, Timika 99963, Indonesia; 2 Mimika District Health Office, Timika 99963, Indonesia; 3 Puskesmas Limau Asri, Mimika District, Timika 99963, Indonesia; 4 Mimika District Penitentiary, Kuala Kencana 99968, Indonesia; 5 Centre for Tropical Medicine, Faculty of Medicine, Public Health and Nursing, Universitas Gadjah Mada, Yogyakarta 55281, Indonesia; 6 Department of Health Policy and Management, Faculty of Medicine, Public Health and Nursing, Universitas Gadjah Mada, Yogyakarta 55281, Indonesia; 7 Menzies School of Health Research, Charles Darwin University, Darwin, NT 0810, Australia; 8 Department of Public Health, Faculty of Medicine, Universitas Sebelas Maret, Kota Surakarta 57126, Indonesia

**Keywords:** tuberculosis, latent tuberculosis, screening, prevention, prison, Indonesia

## Abstract

Incarcerated people have been reported to have higher rates of tuberculosis (TB) than the general population. However, TB is rarely reported among incarcerated people in correctional facilities in Mimika District, in Central Papua Province of Indonesia. This study aims to describe the outcomes of comprehensive screening and treatment of TB disease and latent TB infection (LTBI) within a prison in Mimika. In response to a newly reported case of TB within a prison, a facility-wide comprehensive screening and treatment program was carried out for both TB disease and LTBI between September 2021 and June 2022. We evaluated the outcomes of the screening intervention, including the number of people found to have TB and LTBI and the number and proportion of people who started and completed TB-preventive treatment at the facility. A total of 403 incarcerated people and facility staff participated in the comprehensive screening program. Ten participants were found to have TB disease, all of whom commenced treatment. LTBI was detected in 256 (64%) participants, 251 (98%) of whom completed TB-preventive treatment. Comprehensive screening revealed a high prevalence of TB disease and LTBI in this prison. Completion of treatment for TB disease and latent TB infection was high. These outcomes suggest a role for routine search–treat–prevent strategies for TB in this setting.

## 1. Introduction

Incarcerated people are at a high risk of being infected with and developing tuberculosis (TB) disease [1,2,3]. The burden of TB among prison populations globally is estimated to be 10-foldhigher than the general population [1]. Incarcerated people face a complex mix of risk factors and vulnerabilities to TB, such as a greater burden of physical and mental comorbidities, undernutrition, smoking and substance misuse, social and financial disadvantage, and marginalization [4]. Further, prisons are often high-risk environments for the transmission of TB to occur, due to the high movement of people into and around the prison system, frequent overcrowding, poor ventilation, and inadequate access to healthcare [4,5]. As a result, TB in prisons is recognized as a priority public health issue [4].

Indonesia is one of the highest TB burden countries globally, with an estimated incidence of 1.06 million cases of TB in 2022, equating to an incidence rate of 385 per 100,000 population. It is estimated that 2.2% of people with newly diagnosed TB and 25% of those with previously treated TB have rifampicin-resistant disease [4]. Despite a substantial increase in the number of notified TB cases in 2022, there remains a large gap in treatment coverage, with approximately one-third of the estimated number of incidence cases going undiagnosed and untreated. Publicly available data on the burden of TB in prisons in Indonesia is lacking; however, given the high TB incidence in the general population, reports of significant overcrowding within the Indonesian prison system [6,7], and comparative data from other countries in Southeast Asia [1,2], it is likely that the burden of TB among incarcerated people in Indonesia is high.

Central Papua Province, located in the east of Indonesia, has a particularly high burden of TB, with an annual TB notification rate of 818 cases per 100,000 population [8]. Over the last decade, there has been a concerted effort to scale up active case finding for TB in Central Papua; however, interventions for this have focused on household contacts of people with TB and people living with HIV [9]. The World Health Organization (WHO) recommends systematic screening for TB disease in prisons and penitentiary institutions [10], as well as consideration of systematic LTBI testing and treatment of prisoners [11]. Screening for TB symptoms is periodically undertaken among people incarcerated within a prison setting in Central Papua, but few new cases of TB have arisen from this routine practice. However, in response to a single case of pulmonary TB that was identified through routine screening, a comprehensive search–treat–prevent strategy was developed to screen the prison population for TB disease and latent TB infection. This study aims to implement and assess the outcomes of this intervention to address TB in a prison in Central Papua Province, Indonesia.

## 2. Materials and Methods

We conducted a cross-sectional study to evaluate the outcomes of a comprehensive search–treat–prevent intervention among incarcerated people and staff at a prison in Mimika, within the province of Central Papua, Indonesia. The facility had a recommended capacity of 266 incarcerated people, who were housed across six male blocks and one female block and included people aged from mid-adolescence through adulthood. The study population included incarcerated people and staff at the facility.

Baseline data on TB screening outcomes were obtained for the period September 2021–March 2022. The comprehensive search–treat–prevent intervention was implemented between April 2022 and June 2022 (intervention period). People eligible to participate in the comprehensive search–treat–prevent intervention were all people incarcerated as well as staff who worked within the facility during the intervention period and consented to participate in screening.

Routine screening for TB before the intervention consisted of periodic symptom screening of the prison population, conducted every three months by staff from the local Puskesmas (government community health clinic), using the WHO four symptom screen [12]. People reporting one or more TB symptoms (current cough, fever, weight loss, or night sweats) were referred for sputum collection and testing for TB in line with local procedures.

**Figure 1:**
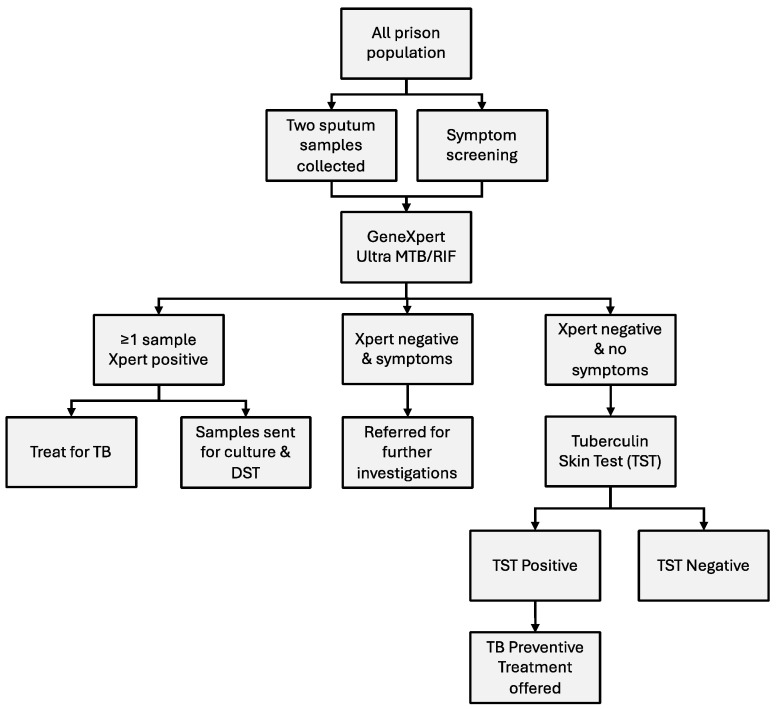
Screening algorithm used during the intervention period.

The first component of the comprehensive search–treat–prevent intervention consisted of educational sessions. The eligible population were all staff and people incarcerated at the facility. These sessions allowed the sharing of information about TB, its transmission, common signs and symptoms, TB treatment and prevention, and the components of the search–treat–prevent intervention. This was followed by obtaining consent and registration of participants. Consenting participants then underwent TB symptom screening which was undertaken by a team of trained TB clinicians from the District Health Service TB program and local Puskesmas, and the collection of two sputum samples for testing by GeneXpert Ultra MTB/RIF^®^ (Cepheid, Sunnyvale, CA, USA) (Xpert), regardless of the presence or absence of symptoms. A diagnosis of TB disease was made by the medical team based on clinical information and GeneXpert results. Samples from GeneXpert positive people were sent to the provincial referral laboratory for confirmatory testing by culture and drug-susceptibility testing. People found to have TB disease were commenced on anti-TB treatment in line with national guidelines (Figure 1).

Participants in whom TB disease was excluded based on the absence of TB symptoms and negative Xpert were eligible for testing for latent TB infection. This was performed using a Tuberculin (Mantoux) skin test (TST). TST results were read 48–72 h following the administration of tuberculin-purified protein derivative, and latent TB infection was defined as induration of ≥10 mm. Those who were found to have evidence of latent TB infection were offered TB-preventive treatment, (Figure 1), which consisted of 12 weekly doses of rifapentine and isoniazid (3HP). Treatment completion was defined as receipt of at least 11 doses of 3HP within 16 weeks of commencing treatment.

During both the baseline and intervention periods, screening, treatment, and preventive treatment data were recorded using the routine TB reporting database. After the intervention period, we extracted data from this database for all members of the eligible population for comprehensive screening, as well as for a baseline period prior to the implementation of the comprehensive screening program. Data were cross-checked and validated by members of the TB case-finding team. We performed data quality checks prior to analyzing the dataset. Data analysis was performed using descriptive statistics to describe the study population. The cascades of care for both TB active case finding and TB-preventive treatment were constructed by calculating the proportion of the total eligible study population proceeding through each milestone of the screening and treatment processes.

We created contingency tables and calculated the Chi [2] statistic to compare outcomes and determine factors associated with latent TB infection among the study population. Statistical significance was determined using a *p*-value of <0.05. Data were analyzed using R Statistical Software (version 4.3.0; R Foundation for Statistical Computing, Vienna, Austria).

## 3. Results

A total of 403 people participated in the comprehensive search–treat–prevent intervention. Of these, 357 (88.6%) were incarcerated people and 46 (11.4%) were staff of the facility. The median age of those incarcerated was 32 years (range: 16–71 years) and 26 years (range: 19–54 years) for staff. The majority of those incarcerated were male (91.3%), whereas staff participating in screening were predominantly female (84.8%) (Table 1).

Prior to the implementation of the comprehensive TB screening activity, within a cohort of 347 incarcerated people, representing the total resident population of the facility during this time, all were subject to TB symptom screening. Among these, 16 people (4.6%) reported TB-compatible symptoms, of whom 13 (81.4%) had a sputum Xpert test completed. All thirteen of them returned a negative Xpert result.

During the implementation period of the comprehensive search–treat–prevent program, there were a total of 432 incarcerated people and staff who were eligible to participate. Among these, 403 (93.3%) underwent screening. Xpert results were available for all those screened. Among those who participated in the intervention, 20.3% reported one or more TB symptoms. This was significantly higher than the proportion reporting symptoms during the baseline period (Odds Ratio: 5.2 (95%CI: 3.1–9.5), *p* < 0.0001). All of those who participated provided at least one sputum sample and Xpert results were available for all participants tested.

**Figure 2:**
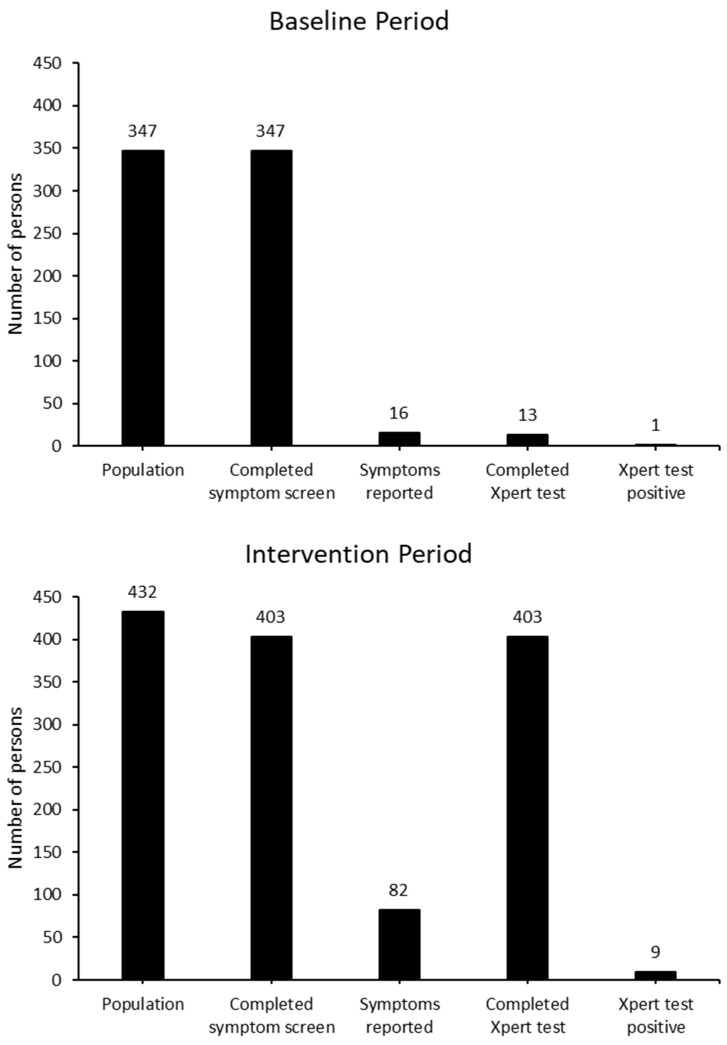
TB screening cascade, baseline period (routine symptom screening) versus intervention period (systematic symptom screening and universal sputum testing).

Following the initial case of TB reported in an incarcerated person during routine screening, nine additional participants tested positive for TB by Xpert during the intervention period, equating to a total crude prevalence among those screened of 2.48% (95%CI: 0.96–4.00%). Among those who returned a negative Xpert result, no additional cases of TB were diagnosed on clinical grounds. Among the ten TB cases detected during the comprehensive screening intervention, all had completed routine symptomatic screening during the baseline period. Five of them had reported TB-compatible symptoms at that time but were not found to have TB through available clinical and bacteriological investigations. All ten people who tested positive for TB successfully completed anti-TB treatment (Figure 2).

**Figure 3:**
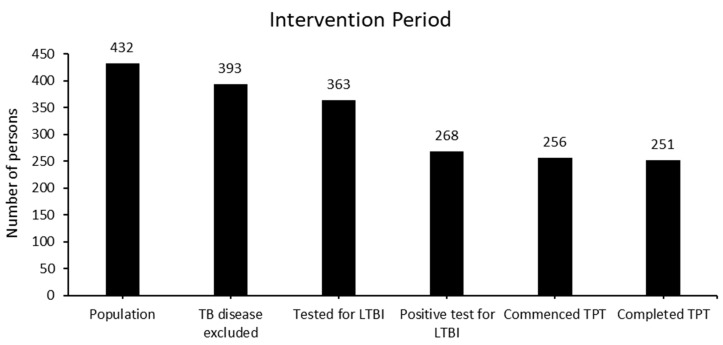
Latent TB infection screening and treatment cascade.

Among the 393 people in whom TB disease was excluded, 363 (92%) completed a TST. A TST positivity of 74% (95%CI: 69.3–78.4%) was observed among the study population. Among those with evidence of LTBI, 96% commenced TB-preventive treatment with 3HP, and 98% of those who commenced treatment successfully completed it, equating to overall treatment completion of 94% of people with evidence of LTBI within the study population (Figure 3). None of those who commenced 3HP reported adverse events that required the discontinuation of their treatment.

## 4. Discussion

We observed a very high prevalence of TB disease within the study setting, equivalent to 4.2 fold the estimated prevalence within the general population of Indonesia [4]. TB prevalence within prisons varies substantially, particularly by region. Our findings are consistent with rates of disease reported in other settings. Regional estimates of the incidence of TB within prisons in Southeast Asia of 1490 and 1550 cases per 100,000 population, derived from meta and systematic analyses of published data, have been previously reported [1,2]. A large proportion of our study population had evidence of LTBI (74%, 95%CI: 69.3–78.4%). Data on the prevalence of LTBI in the general population in Indonesia are limited; however, in comparison with LTBI rates reported in other prison settings, our findings are comparatively high. A meta-analysis of the prevalence of LTBI among 60,808 prisoners found a prevalence estimate of LTBI of 44.4% (95%CI: 30.0–59.8%) [3].

In comparison to the baseline period, a significantly higher proportion of the study population reported one or more TB symptoms during the intervention period. Several factors could explain this discrepancy. Some participants who reported symptoms during the intervention period may have had an onset of symptoms that occurred after the baseline period. However, it is likely that the incorporation of TB education prior to screening during the intervention period may have prompted participants to recognize and report TB symptoms. We did not assess the impact of providing education on TB-related knowledge of participants, however, studies in other settings have demonstrated that short educational interventions can improve knowledge of TB [13,14].

A limitation of TB screening within our study setting is that the use of X-ray was not included in the screening algorithm—both in the baseline and intervention periods. This was due to the unavailability of X-rays within the prison setting. Data from national TB prevalence surveys and other sources have demonstrated that between 30% and 50% of people with TB are asymptomatic [15,16]. Thus, we expect that routine symptom screening, such as that undertaken during the baseline period, is likely to miss a substantial proportion of TB cases. To address the lack of availability of X-rays during the intervention, our screening algorithm utilized universal Xpert Ultra testing, regardless of the presence or absence of symptoms. This has been previously found to be a sensitive, yet cost-efficient strategy for TB screening in a prison setting [17]. Despite the high sensitivity of Xpert Ultra, there is a risk of false-negative results, which is higher among some sub-groups such as those with paucibacillary disease or people living with HIV [18]. In the context of screening and treating TB disease and LTBI, the availability of chest X-ray to exclude TB disease prior to initiating TB-preventive treatment may be considered preferable. Incorporating chest X-rays, including the use of computer-aided detection (CAD) software, along with symptom screening, as an initial screening to determine eligibility for Xpert testing may help to reduce the number of Xpert cartridges required and the burden on TB laboratories [19].

We achieved high uptake and completion of TB-preventive treatment, using the short 3HP regimen. A systematic review of treatment of LTBI in incarcerated people published in 2023, found results from 11 studies, most of which were published in low-TB burden countries. Treatment completion ranged from 26% to 100%, with transfer to other facilities and loss to follow-up being common reasons for discontinuation of treatment [20]. Our findings suggest that LTBI can be treated in prison settings, even in high-burden, low-resource settings. Similar findings of a high burden of disease and infection and good uptake of TB-preventive treatment have been observed in other settings [21].

In 2024, there were 273,390 people in the prison population of Indonesia [22]. Given the large size of this population group and the high burden of undiagnosed and untreated TB disease and LTBI, it represents a population group which may benefit significantly from active case finding and TB prevention strategies.

## 5. Conclusions

Strategies to scale up active case finding and TB prevention are needed in Central Papua Province of Indonesia to achieve progress towards global targets to end TB targets. Our comprehensive search–treat–prevent intervention identified a high prevalence of both TB disease and LTBI in a prison setting where TB disease had not been previously identified and screening for LTBI had not been attempted. High uptake and completion of treatment for both TB disease and LTBI were achieved. Our findings suggest that such an intervention is a feasible approach to identifying and treating TB in correctional facilities. Additional research is needed to determine the impacts of screening on TB epidemiology in prisons, the acceptability of screening among incarcerated people, cost-effectiveness, and optimal screening algorithms for TB screening in this setting.

## Figures and Tables

**Table 1:** Characteristics of participants in the comprehensive prison screening program, Mimika District, 2022.

		Incarcerated		Staff	
Characteristics		N	%	N	%
Sex	Male	326	91.3	7	15.2
	Female	31	8.7	39	84.8
Ethnicity	Papuan	70	19.6	10	21.7
	Non-Papuan	287	80.4	36	78.3
Age group	≤25 years	91	25.5	20	43.5
	26–31 years	79	22.1	13	28.2
	32–39 years	95	26.6	8	17.4
	>40 years	92	25.8	5	10.9
Area of facility	Area 1	31	8.7		
	Area 2	26	7.3		
	Area 3	40	11.2		
	Area 4	29	8.1		
	Area 5	67	18.8		
	Area 6	84	23.5		
	Area 7	80	22.4		
Total		357	88.6	46	11.4

## Data Availability

Due to data privacy concerns, data are not made publicly available. However, reasonable data requests may be granted through contacting the corresponding author.
